# Entropy-Based Strategies for Rapid Pre-Processing and Classification of Time Series Data from Single-Molecule Force Experiments

**DOI:** 10.3390/e22060701

**Published:** 2020-06-23

**Authors:** Denis Horvath, Gabriel Žoldák

**Affiliations:** Center for Interdisciplinary Biosciences, Technology and Innovation Park, University of Pavol Jozef Šafárik, Jesenná 5, 041 01 Košice, Slovakia; gabriel.zoldak@upjs.sk

**Keywords:** single-protein dynamics, entropy-based classification, signal pre-processing

## Abstract

Recent advances in single-molecule science have revealed an astonishing number of details on the microscopic states of molecules, which in turn defined the need for simple, automated processing of numerous time-series data. In particular, large datasets of time series of single protein molecules have been obtained using laser optical tweezers. In this system, each molecular state has a separate time series with a relatively uneven composition from the point of view-point of local descriptive statistics. In the past, uncertain data quality and heterogeneity of molecular states were biased to the human experience. Because the data processing information is not directly transferable to the black-box-framework for an efficient classification, a rapid evaluation of a large number of time series samples simultaneously measured may constitute a serious obstacle. To solve this particular problem, we have implemented a supervised learning method that combines local entropic models with the global Lehmer average. We find that the methodological combination is suitable to perform a fast and simple categorization, which enables rapid pre-processing of the data with minimal optimization and user interventions.

## 1. Introduction

Only the set of averaged characteristics can be captured by bulk experimental methods, which significantly limits our understanding of the heterogeneity of molecular states. On the other hand, single-molecule techniques provide deep insights into the complex dynamics of individual molecules [1]. Namely, the detection of various molecular sub-states, conformations as well as interstate transformations is one of the main benefits of single molecule techniques [2].

Hence, in general, single-molecule techniques offer the possibility to characterize molecular heterogeneity and to quantify a number of sub-states, interconversion rates, and their occurrences. A development of advanced approaches is essential to enhance experimental resolution, which is needed for describing rare, low-populated states of molecules. It is important to note that in biological systems, such low-populated rare sub-states can have profound effects. For example, the rare, low-population infectious states of prion protein PrP are highly crucial as they act as a nucleated seed that recruits native PrP into fibrils that ultimately contribute to amyloid disease [3]. Importantly, single-molecule force spectroscopy of prion protein PrP has identified and characterized low populated rare misfolded states [4]. This example demonstrates the power of single-molecule techniques to detect relevant low-populated rare sub-states. Naturally, capturing and detecting low-populated rare sub-states using single-molecule techniques is experimentally challenging; it requires extensive time-series data collection, selection, and categorization. Additionally, several complications arise from the presence of inactive, latent states. For example, single-molecule force spectroscopy utilizes relative high laser powers, which can lead to dormant states that need to be identified and distinguished from other molecular states. At the moment, such states can be filtered out only after user intervention during the data pre-processing analysis. In other words, single-molecule detection of heterogeneous states needs to sample, to analyze, and to handle extensive number of datasets. However, large data handling can be very laborious and time consuming. For example, more than 8 h are needed for a single investigator to analyze and visualize time trajectories of 100 molecules under a gross assumption that 5 min are required for data loading, visualization, and inspection of a single time trace.

Thus, to evaluate more extensive time-series data efficiently, there is a strong need for the development of methods to allow fast, special purpose tailor-made pre-processing of the time series samples. The effort to quantify the intrinsic information in the data is a crucial general principle of the pre-processing method described here.

The principal concept related to information content is entropy. It is natural, therefore, that the method of choice, that we are mainly dealing with is linked to the entropy variants. Given that the experimental signal is not stationary, calculations of the entropy for its small time sections should be used. In general, two basic forms of implementation are devoted to the concept of entropy in time series analysis tasks. The complexity analysis of dynamic systems is often based on the Kolmogorov-Sinai entropy [5,6], also known as metric entropy. It relies on the division of phase space into hypercubes. While this method offers a well-defined information-based predictability assessment, it faces the fundamental problems that have arisen concerning data processing. Efforts for less demanding processing, especially of biologically relevant data, were subsequently reflected in the design of approximate entropy (ApEn) and later in the modified sample entropy variant (SampEn) [7,8]. Some of the shortcomings of these approaches have been addressed by the adjustments contained in the multiscale description (MSE) [9].

In contrast to these procedures, studying the properties of the molecules in an experiment in which the data provided focus on the properties of the individual molecules imposes different requirements on the evaluation and pre-processing of data. In our case, therefore, to ensure compatibility, the entropy estimates are tracked via time-truncated local histograms. This aspect of the description is highly consistent with the use of Hidden Markov Models (HMM) used to characterize structural changes and dynamics of biomolecules [10]. The methods we proposed to use is an adaptive approach to the formation of histogram bins. Specifically, there is a focus on implementing entropy models where free parameters can be integrated into optimization or learning paradigms. In particular, we refer to the entropy forms of Tsallis [11,12]. The analogous adaptation can also be done by analysis of Rényi entropy [13].

The paper structure is as follows. We start with the description of the experimental methods and methodologies used in Section 2. Section 2.1 deals with description of the methodology of optical tweezers and character of the data used. The model details such as corresponding structure of histograms, related entropy evaluations and the specific role of the Lehmer averages are explained in Section 2.2. It is a specific local form used for the scrolling time window. The evaluation of 63 data samples of PrP in Section 3 follows. The comparison with other classification methods which are not related to Tsallis entropy is presented in Section 3.1. Some other pre-processing options regarding integral forms of indicators are included, as well as ideas for further improvement and relations to the statistical testing are provided in Section 3.2. Finally, in the conclusions we present possible avenues for further research, especially those that are in line with HMM.

## 2. Materials and Methodology

### 2.1. Experiments, Protocols, Signal Detection

All experiments were performed using a custom-built, high-resolution back focal plane detection optical tweezers setup, as published previously [14]. For details on experimental procedures, see [15,16]. Briefly, the E. coli Hsp70 nucleotide-binding domain protein construct was genetically modified to serve cysteine residues for the attachment of the required double-stranded DNA handles [15,16]. These DNA handles carried the modifications on each end to ensure coupling to the one μm functionalized beads. The beads could be trapped in our optical tweezers setup and manipulated in a so-called passive mode (for details see [17]). Trapped beads were calibrated according to a method [18], trap stiffness was between 0.25 to 0.30 pN/nm. Signals were acquired for a 10–30 min at a sampling rate of 30 kHz. For the data analysis, the difference between both signals was calculated after the experiment to increase the signal-to-noise ratio [14].

The signals were corrected for a cross-talk for both due to the depolarization and proximity of the beams. For the final analysis, long time traces were analyzed after the resampling to 10 kHz. Glass beads (1μm in diameter; Bangs Laboratories, Inc., Fishers, IN, USA), which were previously covalently functionalized with a digoxigenin Fab fragments (Roche), were mixed with protein–DNA constructs. After the addition of streptavidin-coated silica beads (1μm in diameter; Bangs Laboratories, Inc.), the protein–DNA–bead mixture was introduced into a flow cell. Measurements were carried out at ∼28 °C in PBS (10 mM phosphate buffer, 2.7 mM potassium chloride, 137 mM sodium chloride, pH7.4, at 25 °C), with an added oxygen scavenger system (26 U/mL glucose oxidase, 17,000 U/mL catalase, 0.65% glucose). During the single-molecule mechanical measurements, trapped beads were brought into proximity to build a bead–DNA–protein dumbbell. Protein–DNA concentrations were adjusted to sparsely cover the beads leading mainly to single-tether formation. The trapping potentials were held at a constant separation to record passive-mode force vs. time traces.

#### Problem Formulation—Data Categories

**Figure 1:**
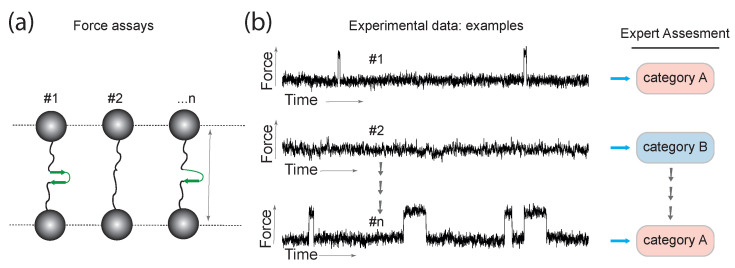
The figure depicts the scheme of the single molecule experiment. Panel (**a**) shows the molecular force assay on a single-molecule level where the elastic responses are generated. The time series responses (tools for probing energy landscapes) followed by a respective expert categorization are illustrated in panel (**b**).

We will now describe the idea of activities and the role of an expert in the classification. The expert will assume that she/he disposes of a set of single-molecule force experiments (see Figure 1). For simplicity, let us consider the experiments generating two types of time series data, i.e., two types (categories) of samples, which are denoted as A and B. For type A (category A), further detailed processing and research is necessary to gain insights into single-molecule kinetics. On the other hand, experiments of type B are considered to be the result of entirely different molecular states (e.g., damaged molecule, or in a transient misfolded state) and will not be further investigated in detail. Still, the counting of such experiments in category B provides numbers for statistical evaluations. Type A (category A) means that the measurement provides only a few discrete molecular states. There are visible transitions between these states. With the type B, the states are not spatially and temporally separated enough or only the molecules resting in a single state, and hence no transitions can be identified.

Only high-quality single-molecule data can provide reliable information on the underlying free energy landscape. Here we show that histogram analysis can play a dual role in the data processing from single-molecule force spectroscopy. Single-molecule data pre-processing, as demonstrated in the presented study, can be included in the beginning of the data analysis pipeline. As our histogram-based pre-processing method energy is general and independent of the underlying energy landscape, the outcoming experimental data in category A can be further processed. There are several ways to extract effective free energy landscapes from single-molecule time series using histogram analysis [19,20]. The procedure identifies a distribution of the observable associated with each local equilibrium state. By assessing how often the molecule visits and resides in a chosen state and escapes from one state to another, their analysis naturally leads to a reconstruction of the free energy landscape. In another approach, the time series of a single intramolecular distance can be analyzed by a network-based method for determining basins and barriers of complex free energy surfaces (e.g., the protein folding landscape).

### 2.2. Measures and Methods of Supervised Classification

In the next we go step by step through the main elements of the classification system described in Section 2.2.1, Section 2.2.2 and Section 2.2.3.

#### 2.2.1. Time Series, Averages, Adaptive Histograms

In compliance with data, we consider time series ${\left\{x\right\}}_{t}$ real-valued subsequent observations $x_{t}$. The experimental conditions do not allow us to assume that observations are uniformly distributed. To make the problem computationally feasible, the situation can be improved by splitting the original signal into smaller parts-time windows. The data is considered to be partially stationary in the respective window. For each window, $t\in [T_{\mathrm{wdn}},T_{\mathrm{wup}}]$, $T_{\mathrm{wup}}-T_{\mathrm{wdn}}=T_{w}=\mathrm{const}$. the local mean values resulting from the iterative evaluation can be obtained as presented in the Algorithm 1.
**Algorithm 1:** Conditional mean values for given time window.
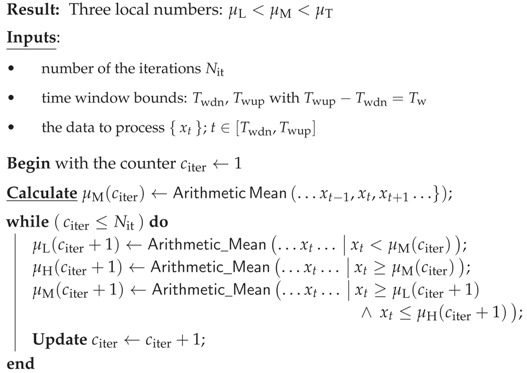


Because histograms change dynamically, the peak heights and valley depths of valleys between different time windows, we have designed a processing method which we called adaptive. In this particular framework, it is envisaged that the shape of the bins can be adapted to immediate situations rather than just inefficiently increasing the number of breaks to achieve a certain level of complexity.

Specifically, we gain adaptability by sustaining a constant number of breaks in a changing position. After the repeated stabilization and iterative improvements of the respective average values, we calculated the respective conditional probabilities

(1) $$\begin{array}{ccc} \pi_{0} & = & \mathrm{Prob}(\;x|\;x\leq \mu_{L}\;)\\ \pi_{1} & = & \mathrm{Prob}(\;x|\;x\in \left(\mu_{L},\mu_{M}\right]\;)\\ \pi_{2} & = & \mathrm{Prob}(\;x|\;x\in \left(\mu_{M},\mu_{H}\right]\;)\\ \pi_{3} & = & \mathrm{Prob}(\;x|\;x>\mu_{H}\;). \end{array}$$

For the sake of simplicity, the values $\pi_{\ldots }$, $x_{\ldots }$, $\mu_{\ldots }$ are not provided with a time stamp. Another rationale for this reduction is that we are concerned of some form of possible window rearrangement at this level, as there is no influence on the outcome. The result can be considered in the form of a elementary histogram with only three adaptive breakpoints $\mu_{L}$, $\mu_{M}$, $\mu_{H}$. Adaptability is essential because data properties can change over time. A well-adapted, concise and substantially reduced histogram can consist of only a few uneven breaks.

#### 2.2.2. Entropy of Histograms

Further considerations are central to the concept of entropy, which is a natural and integral and universal part of the probabilistic description. The entropy measure does not highlight some of the details of the histogram, but reflects the level of organization required for the success of preprocessing. The preprocessing information only becomes relevant when entropy values are affected by specific control parameters. If the internal parameters (meta-parameters) of the data mapping model are incorporated into the learning process, some of their instances may be more suitable for certain types of processed data. The T-entropy introduced by Tsallis [11,21] is an ideal parametric candidate that can provide distinguishable inter-class separation in the output values. Its form

(2) $$S_{T}\left(q_{T}\right)=\frac{1-\sum_{j=0}^{3}\pi_{j}^{q_{T}}}{q_{T}-1}\;$$

uses the real parameter $q_{T}$. An alternative to this is, for example, the Reny’s form of the entropy.

**Figure 2:**
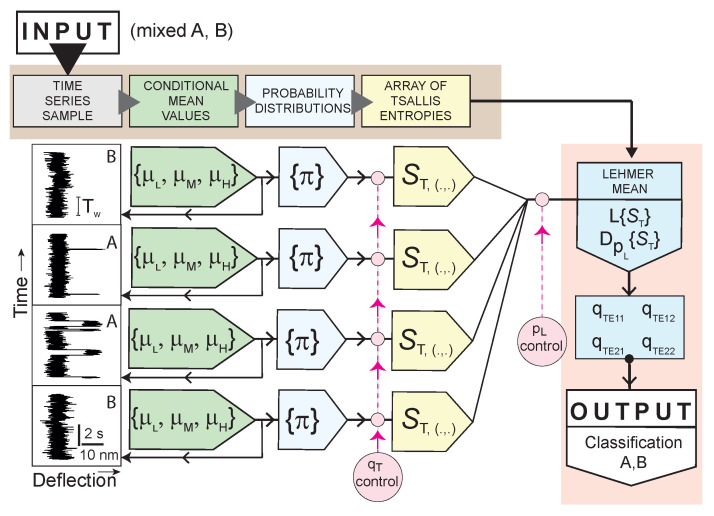
The scheme shows how partial blocks are organized into an overall algorithm. It is a kind of nonlinear filtering of the input time series. In a hypothetical inference process, a comparison can be made with the transformed elements sampled from data categories A or B. The result of the design is a non-linear filter–classifier, which conceptually relies on the need for a supervised learning phase.

It should be noted that we are not introducing the entropy of the entire time series, instead, our proposal is T-entropy suggested for different time windows. Now it is useful to look at the overall computational model depicted in Figure 2, which briefly describes the structure of data processing flows as well as the organization of the time windows. Each data treatment is based on the exchangable collection of T-entropy values constructed for the constant $T_{w}$. The selection of $T_{w}$ uniquely determines the number of the non-overlapping windows $n_{w}$ = floor (Number_of_time_series_tics/$T_{w}$). Of course, the overlaps are not ignored as they provide additional statistical information that partially eliminates the reliance on selecting the initial time window. The overlap effect is characterized by the independent positive integer $n_{\mathrm{ws}}$ (see details described by the Algorithm 2). The method described above transforms the original data series into 2D array of the local T-entropies

(3) $$\begin{array}{c} \begin{array}{cccc} S_{T,\left(1,0\right)} & S_{T,\left(1,1\right)} & \ldots , & S_{T,\left(1,n_{\mathrm{ws}}-1\right)}\\ S_{T,\left(2,0\right)} & S_{T,\left(2,1\right)} & \ldots , & S_{T,\left(2,n_{\mathrm{ws}}-1\right)}\\ \ldots & \ldots & \ldots & \ldots \\ S_{T,\left(n_{w}-2,0\right)} & S_{T,\left(n_{w}-2,1\right)} & \ldots , & S_{T,\left(n_{w}-2,n_{\mathrm{ws}}-1\right)} \end{array} \end{array}$$

with the structure

(4) ST,(indexofnon-overlapingwindow,indexcharacterizingoverlap).

The statistics of $S_{T,(.,.)}$ became evidently non-Gaussian due to constraints and therefore ceased to be suitable for simple characterization by mere arithmetic means.
**Algorithm 2:** Lehmer mean of set of entropy values.
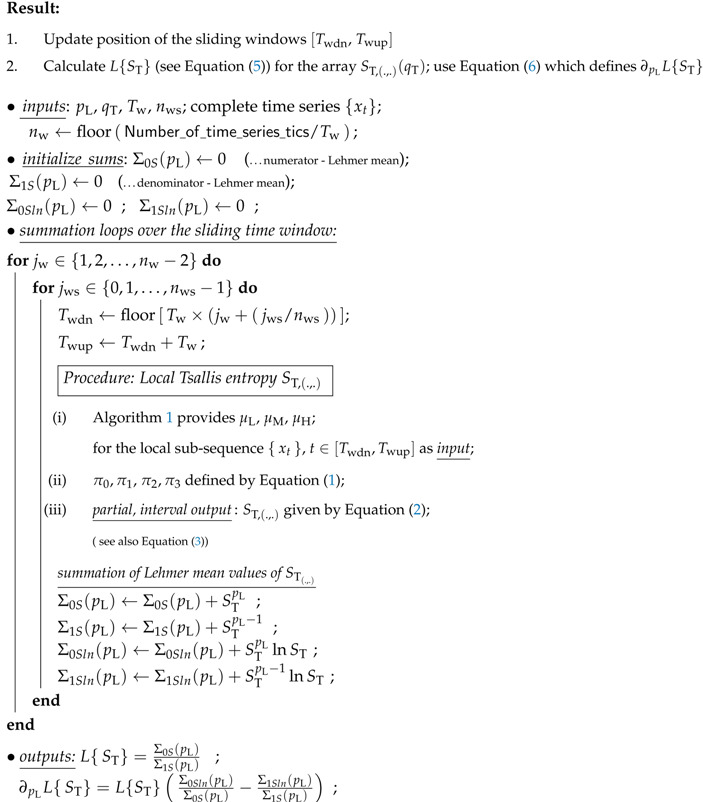


Let us now turn to the main physical properties of the data that we want to identify and quantify. Their details and manifestations fall within the scope of the classification, which will depend upon the decisions of the specialist. The classification process in our specific application means that the sample is assigned to one of the defined classes (A or B). We believe that after a series of transformations we make, there will be a continuous separation zone between A and B that will be sufficiently wide enough.

Of course, more experiments with control parameters should point to the potential for higher sensitivity of our transformation in data processing. Our concept evolved mainly from the preliminary requirement that the transformation of a sample with bimodality or multimodality is adequately separated from the transformation of a sample without these statistical characteristics. However, we did not follow these requirements strictly below because we do not want to focus too much on a specific pattern. Instead, we prefer a more general approach. T-entropy [11] could be ideal for this purpose. In the following, we assume that T-entropy on relatively small scales or its generalized mean values (large scales) could be effective in the classification process. We realize that what we are now proposing is a more abstract, not a definitively valid strategy, but that numerical analysis can ultimately reveal knowledge and bring (parametric) improvements that can be applied in the upcoming learning and optimization process. The numerical schemes we use here are in principle consistent with supervised learning methods. We note that we have attempted several approaches, but only a few attempts have worked well, leading to the basic empirical version that we publish in this work.

Nevertheless, let us also mention details regarding the numerical experiments with classification, which initially do not produce satisfactory results. For example, an alternative direct calculation of the so-called Sarle’s b ($\;b_{\mathrm{Sarle}}$), which is typically used to detect bimodality [22] (based on combination of kurtosis and skewness), did not provide a proper segregation of A and B and was therefore not a valid distinguishing feature for sets A and B. An obvious explanation is that the value of the $b_{\mathrm{Sarle}}$ fluctuates considerably along time series values. For example, a particular window may not necessarily be in the correct place to extract the entire statistically representative sample. In Section 3.1 we present several examples of variants for which the averaging method is of high relevance. An interesting alternative to the conventional approach to $b_{\mathrm{Sarle}}$ is described in Section 3.1.3.

#### 2.2.3. Long-Term Transformation into Entropic Systems with Related Lehmer Means

Obviously, multimodality and bimodality can reduce entropy compared to uniform distribution of states. However, this also applies to individual isolated distribution peaks that are not of interest. Paradoxically, therefore, entropy may seem to be a relatively general and to some extent imperfect indicator, which may not suit the needs of experts. In other words, this seems to be a weak alternative to identifying detailed changes in each distribution. On empirical basis, the fundamental premises regarding the entropy series will be sufficient for a given classification, and the entropy will be effective enough to enable rapid classification of sample types.

Let’s turn to the details now that we need a generalized averaging of the entropy series. Any candidate averaging method that seeks to achieve a sufficient separation of A and B should take into account the fact that not all entropy data should be considered with the same weight. For example, the Lehmer mean can characterize the asymmetric distributions of $\left\{S_{T}\right\}$ values reliably. To be more explicit, when consider the set $\left\{X_{j}\;\right|$ $\;X_{j}$∈$R^{+}\}$ the Lehmer mean [23,24,25,26] is given by $L\left\{X\right\}\left(p_{L},.\right)=$ $\sum_{j}\;X_{j}^{p_{L}}/\sum_{j^{\prime }}X_{j^{\prime }}^{p_{L}-1}$. There is, of course, freedom of assessment samples using the weights $∼x_{j}^{\left(p_{L}-1\right)}$ depending on the $p_{L}\in R$ parameter.

The above framework helps us to create a particular mean of the entropy sequence, equipped with a variety of window indices. The entropy events collected according to the scheme from Equation (3) lead to the mean

(5) $$L\left\{S_{T}\right\}\left(p_{L},q_{T}\right)=\frac{\sum_{j_{w}=1}^{n_{w}-2}\;\sum_{j_{\mathrm{ws}}=0}^{n_{\mathrm{ws}}-1}\;\;{\left(S_{T,\left(j_{w},j_{\mathrm{ws}}\right)}\left(q_{T}\right)\right)}^{p_{L}}}{\sum_{j_{w}^{\prime }=1}^{n_{w}-2}\;\;\sum_{j_{\mathrm{ws}}^{\prime }=0}^{n_{\mathrm{ws}}-1}\;\;{\left(S_{T,\left(j_{w}^{\prime },\;j_{\mathrm{ws}}^{\prime }\right)}\left(q_{T}\right)\right)}^{\left(p_{L}-1\right)}}.$$

Since we do not know yet which component of the recognition and classification system will be more productive in terms of the projected data, we are also interested in the derivative

(6) $$\begin{array}{c} {\hat{D}}_{p_{L}}\;L\left\{S_{T}\right\}\equiv \partial_{p_{L}}L\left\{S_{T}\right\}=L\left\{S_{T}\right\}\left(\frac{\sum S_{T,(.,.)}^{p_{L}}\ln S_{T,(.,.)}}{\sum S_{T,(.,.)}^{p_{L}}}-\frac{\sum S_{T,(.,.)}^{\left(p_{L}-1\right)}\ln S_{T,(.,.)}}{\sum S_{T,(.,.)}^{\left(p_{L}-1\right)}}\right). \end{array}$$

Here we have intentionally omitted the sum information used in $S_{T,(.,.)}$ (see Equation (5)). More specifically, it would be helpful at this point to grasp the details of the information gathered. For this purpose, the Scheme Algorithm 2 is provided, to give details of how partial contributions are summed up to determine the Lehmer mean values.

In order to differentiate inputs using various techniques of the filtering, we have implemented two entropy-based weighing versions

(7) $$\begin{array}{c} w_{1}\equiv L\left\{S_{T}\right\}\;,\;w_{2}\equiv {\hat{D}}_{p_{L}}L\left\{S_{T}\right\}\;. \end{array}$$

Their effectiveness for a given type of data will be directly examined and commented in the numerical part of the paper. Subsequently, these alternatives were involved in the introduced here system of the effective Tsallis indices

(8) $$q_{\mathrm{TE}yz}\equiv {\left[\frac{\int_{q_{m}}^{q_{M}}dq_{T}^{\prime }\;{\left(q_{T}^{\prime }\right)}^{z}w_{y}\left(q_{T}^{\prime }\right)}{\int_{q_{m}}^{q_{M}}dq_{T}^{\prime \prime }\;\;w_{y}\left(q_{T}^{\prime \prime }\right)}\right]}^{\frac{1}{z}}\;,\;\mathrm{for}\;\;\;\;\;\begin{array}{c} y\in \{1,2\},\\ z\in \{1,2\}. \end{array}$$

In the applications, we limit ourselves to $1<q_{m}\leq q_{M}$ region. In such case we do not need to go through the singular point $q_{T}=1$ (although the singularity is removable). Factor 1/z represents an attempt to “power z compensation” in its essence. We used only $q_{\mathrm{TE}yz}$ (z>0) for the four variants of y,z in the implementation of the proposed method. Of course, the use of very small z should be avoided because of a poor separation effect expected. Regarding the order O(.) of the output we have $O(q_{\mathrm{TEyz}})$ = O$[({\tilde{w}}_{y}/$${\tilde{\tilde{w}}}_{y}{)}^{1/z}]$ $O(\tilde{q_{T}})$, where ${\tilde{w}}_{y}$ and ${\tilde{\tilde{w}}}_{y}$ are two independent mean estimates ${\tilde{w}}_{y}∼{\tilde{\tilde{w}}}_{y}$, thus $O\left({\tilde{w}}_{y}\right)=O\left({\tilde{\tilde{w}}}_{y}\right)$. In addition, let ${\tilde{q}}_{T}$ ∈ $\left[q_{m},q_{M}\right]$ is some representative value which characterizes the interval $\left[q_{m},q_{M}\right]$. Assuming that the choice of $q_{M}$ supports $O({\tilde{q}}_{T})$ = $O(q_{M})$, we have $O\left(q_{\mathrm{TEyz}}\right)=O\left({\tilde{q}}_{T}\right)$. Thus, with the limitations on $q_{T}$, the constraints on $q_{\mathrm{TEyz}}$ are produced. The assumption beyond Equation (8) is that the corresponding $q_{\mathrm{TEyz}}$ indicator provides values of the expected order. This also implies the standardization. The reason for this is that construction is subordinated to Tsallis concept where $q_{\mathrm{TE}yz}$ is by some convolution interlinked to $q_{T}$. Let us repeat again for a better understanding that $q_{\mathrm{TEyz}}$ characterizes the whole time series.

While predictions of $q_{T}$ are not directly included into the underlying theories, many scientific works assume that $q_{T}$ is near the Boltzmann limit $q_{T}\to 1$. As we will demonstrate in the results section, this also applies to the effective version of the parameter with the weights $w_{1}$, $w_{2}$. Although the methodology we are discussing can in principle provide information on a macroscopic statistical property called non-extensivity, it is not clear what happens when the series is processed by Lehmer averaging. Therefore, no attention is paid to this particular issue in the paper.

## 3. Numerical Results

**Figure 3:**
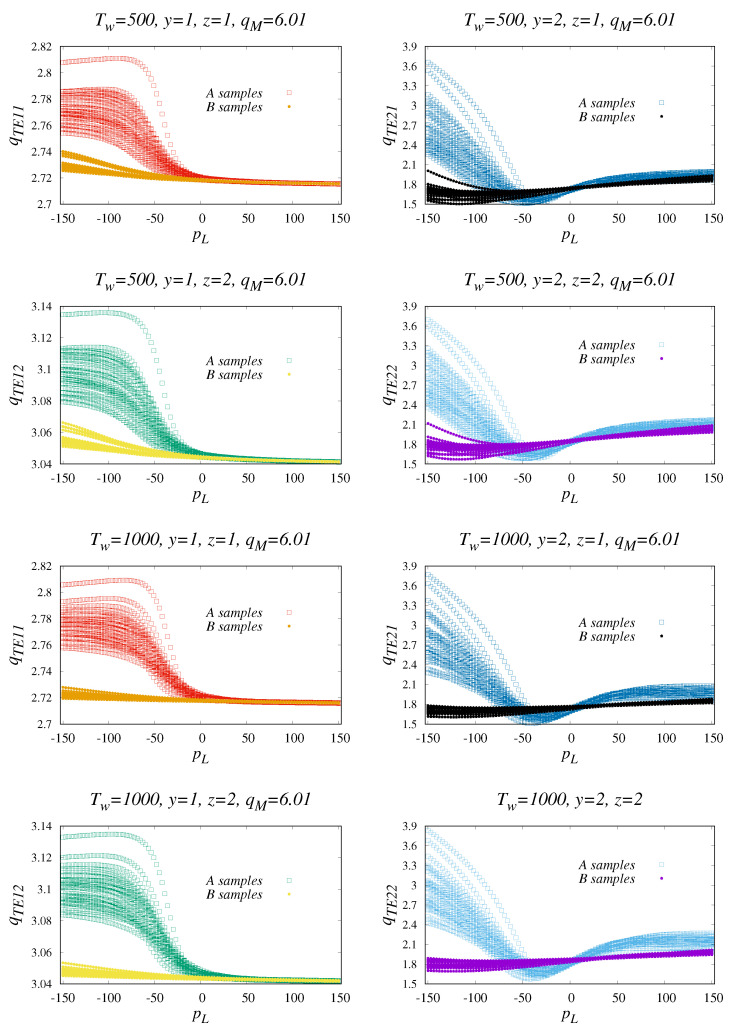
The $p_{L}$ dependencies of $q_{\mathrm{TEyz}}$ obtained for 32 (category A) plus 31 (category B) independent time-series samples. The results do not change too much with $T_{w}$. The panels also include four cases of $q_{\mathrm{TE}yz}$ according to Equation (7). We can see that there is an effective area of negative $p_{L}$ where the classification becomes clear. The projections of experimental samples can be improved by means of z=2 choice.

For the purposes of analysis, we have chosen the following parameter values $N_{\mathrm{it}}=6,$ $n_{\mathrm{ws}}=8$. There are also three primary alternatives $T_{w}=500,$ 1000,2000 which we will later justify by examining the $T_{w}$ dependencies. The behaviour of $q_{\mathrm{TEyz}}$ as a function of $p_{L}$ is depicted on the partial plots of Figure 3. The common basis for the simulation is the use of the boundaries $q_{m}=1.01$ and $q_{M}=6.01$ (see Equation (8)). As checked by our preliminary studies, the efficiency of the separation A and B is highly determined by a sufficiently large $q_{M}$ choice. Initially, we approximated the quadrature by the summation over 1000 evenly spaced nodes. However, we later revealed that numerical quadrature based only upon 10 rectangular sampling of $q_{T}$ not only reduce the calculation load by a factor of 100 but also allow the separation of A and B to be preserved. Exact integration in the sense of Equation (8) is therefore not necessary. In our computational approach, we deal with a quick estimate by means a strongly diluted integration grid (over $q_{T}$). Note that there is a parallel with experimental data analysis that only uses a selection of several different exponent of different regimes using Tsallis distribution [27].

The detailed calculations of $p_{L}$ have been done for three alternatives $T_{w}\in \left\{500,\;1000,2000\right\}$ that offer qualitatively the same result. We are not providing results for the last value here for reasons of redundancy, as there is no significant qualitative impact. We will explain later why the $T_{w}$ performance comparison leads to a benefit of $T_{w}\in$ {1000,2000} variants. (According to the redundancy, there is no figure for $T_{w}=2000$, because there are no qualitatively new effects in the analyzed scenarios). The partial plots of Figure 3 are organized according $T_{w}$ and the choices of $w_{1}$ and $w_{2}$:$w_{1}$ (case y=1), $w_{2}$ (case y=2), and z=1, z=2 (see Equation (7)). As one can see, the use of different weights and various intervals of $p_{L}$ changes the separation effects of A and B. For instance, y=1 admits the substantial separation for the control parameter $-150<p_{L}<-50$. On the other hand, there is no change in the variant y=2 (i.e., for $w_{2}∼{\hat{D}}_{p_{L}}$) but there might be hope in the $-150<p_{L}<-100$ domain.

**Figure 4:**
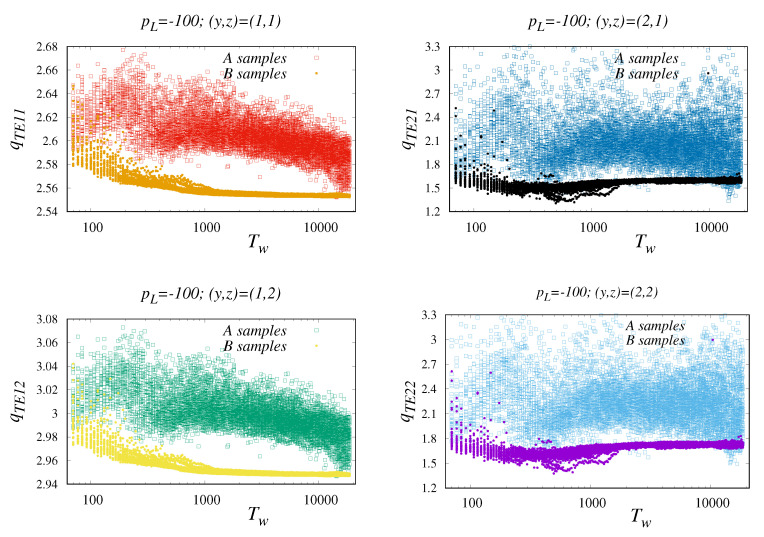
An analysis of the extent to which projections $q_{\mathrm{TEyz}}$ of A, B are separated. The panels in the figure show the $T_{w}$ dependencies of four $q_{\mathrm{TEyz}}$ indicators. Each point on the graph represents output of a separate, parameter-dependent treatment of a single time series. The upper and lower separability bound is better in the z=1 case. For example, we see that the separability scheme works above $T_{w}=500$.

However, how does the size of the window affect the separation into A, B? Obviously, not all window sizes are the source of appropriate solutions. Systematic results from $T_{w}\in \left[0,\;1800\right]$ are summarized in Figure 4 for four combinations y, z as well as for constant $p_{L}=-100$. Prior to these calculations, we verified that above $p_{L}=-50$ the separation between A and B is blurred. In addition, somewhere above $T_{w}=2000$, the results are burdened by considerable diversification and specimen specificity. Another extreme of classification is the small $T_{w}$ domain (for given data, say $T_{w}<200$). This provides very good statistical estimates of averages, but determined only on the basis of a series of significantly biased local entropy.

### 3.1. Comparison of Methods for Specific Time-Series Classification

The purpose of this subsection is to show the broader context and specific comparison between methods. The scope and proposals of comparison are based on the following principles and motivations:

1. the evaluation with the goals to emphasize the gains within the framework of applicability;
2. the design of new potential classifiers with unified and specific mathematical structure;
3. the comparison of new and previously established classification schemes;
4. the identification of the proper parameters (meta-parameters) that are useful for the classification.

Three other indicators are used to compare with Tsallis-based strategies. On the one hand, although the new effective indicators focus on specific aspects, their common feature is the use of the Lehmer average.

#### 3.1.1. Classification Adapted from Kullback–Leibler Form

To adapt our attempts to one of the more traditional approaches of classification, we let ourselves be inspired by the concept of difference and dissimilarity. Therefore, in one of our alternative proposals we favor the use of Kullback–Leibler form.

Let us consider a problem-specific form of Kullback–Leibler divergence

(9) $$S_{\mathrm{KL}}\left(\theta_{\mathrm{KL}}\right)=\sum_{k=0}^{3}\pi_{k}\ln\frac{\pi_{k}}{\pi_{\mathrm{ref},k}\left(\theta_{\mathrm{KL}}\right)}$$

measuring the difference between the original ${\left\{\pi_{k}\right\}}_{k=0}^{3}$ and the symmetric reference distribution

(10) $$\begin{array}{ccc} \pi_{\mathrm{ref},0}\left(\theta_{\mathrm{KL}}\right) & = & \pi_{\mathrm{ref},3}\left(\theta_{\mathrm{KL}}\right)=\frac{\theta_{\mathrm{KL}}}{2},\\ \pi_{\mathrm{ref},1}\left(\theta_{\mathrm{KL}}\right) & = & \pi_{\mathrm{ref},2}\left(\theta_{\mathrm{KL}}\right)=\frac{1}{2}\left(1-\theta_{\mathrm{KL}}\right) \end{array}$$

controlled by the free scalar “homotopy” parameter $\theta_{\mathrm{KL}}\in \left[0,1\right]$. The reference distributions are consistent with the constraint $\sum_{k=0}\pi_{\mathrm{ref},k}=1$. It is obviously assumed that $\left\{\pi_{k}\right\}$ and $\left\{\pi_{\mathrm{ref},k}\left(\theta_{\mathrm{KL}}\right)\right\}$ are from the same probability space. We have checked and confirmed that the choice of symmetric ${\left\{\pi_{\mathrm{ref},k}\left(\theta_{\mathrm{KL}}\right)\right\}}_{k=0}^{3}$ could provide a good approximation of ${\left\{\pi_{k}\right\}}_{k=0}^{3}$.

The parametric form dependent on $\theta_{\mathrm{KL}}$ is suggested to play role similar to $q_{T}$. To be consistent with the previous classification by means of $q_{\mathrm{TE}11}$, we proposed

(11) $$\theta_{\mathrm{KLE}}\equiv \frac{\int_{\theta_{m}}^{\theta_{M}}d\theta_{\mathrm{KL}}^{\prime }{\theta^{\prime }}_{\mathrm{KL}}L\left\{S_{\mathrm{KL}}\right\}\left(p_{L},\theta_{\mathrm{KL}}^{\prime }\right)}{\int_{\theta_{m}}^{\theta_{M}}d\theta_{\mathrm{KL}}^{\prime \prime }L\left\{S_{\mathrm{KL}}\right\}\left(p_{L},\theta_{\mathrm{KL}}^{\prime \prime }\right)}\;.$$

In addition to testing by means of $S_{\mathrm{KL}}\left(\theta_{\mathrm{KL}}\right)$, we work with symmetrized form $S_{\mathrm{KL},\mathrm{sym}}=$ $S_{\mathrm{KL}}+$ $S_{\mathrm{KL}}{|}_{\left[\left\{\pi \right\}\to \mathrm{replace}\to \left\{\pi_{\mathrm{ref}}\right\},\;\left\{\pi_{\mathrm{ref}}\right\}\to \mathrm{replace}\to \left\{\pi \right\}\right]}$. Then, analogously as in the case of Equation (11), we defined $\theta_{\mathrm{KLE},\mathrm{sym}}$. Obviously, the symmetry achieved by the exchange of distributions brings the classification process much closer to the concept of distance.

**Figure 5:**
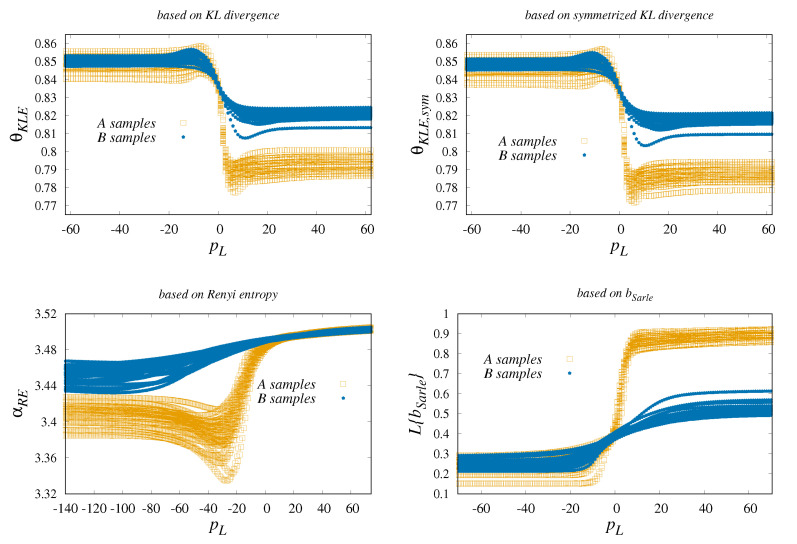
The differences in the alternative forms of the classification are subject to Lehmer selective averaging. Some subtle differences between asymmetric and symmetrical KL divergence are visible. In the case of $\theta_{\mathrm{KLE}}$, $\theta_{\mathrm{KLE},\mathrm{sym}}$, the integration is performed for boundaries $\theta_{m}=0.6$, $\theta_{M}=0.9$. The classification derived from Renyi’s entropy is formulated in terms of $\alpha_{\mathrm{RE}}$ with $\alpha_{m}=1.01$ and $\alpha_{M}=6.01$. The problem of Sarle’s b is revisited. We found that significant positive changes in the classification performance occur with the intervention of Lehmer averaging (the transformation of data to average is denoted by $L\{\;b_{\mathrm{Sarle}}\}$). All depicted examples show the importance of the specific selection of $p_{L}$. Surprisingly, with the exception of $\alpha_{\mathrm{RE}}$, segregation of A from B requires relatively high $p_{L}$.

The numerical results obtained for $\theta_{\mathrm{KLE}}\left(p_{L}\right),\theta_{\mathrm{KLE},\mathrm{sym}}\left(p_{L}\right)$ are shown in Figure 5. They indicate that symmetrization does not provide remarkable differences in the outputs. In addition, there is some robustness in the process of integration. The use of many simulation cycles shows that the choice off $[\theta_{m},$ $\theta_{M}]$ is less important for the global quality of the classification. This is in part due to observed fact that specific regions of $p_{L}$ may improve the accuracy classification process.

The classification based $\theta_{\mathrm{KLE}}$ is freely inspired by the nearest centroid classification method (see, e.g., application in protein detection [28]). The method is based on the premise of distance from the positions of centroids. Inspired by this approach, we have used parametrized reference distributions instead of centroid to define the possible neighbors. The concept of distance, albeit in probability space, remains the basic determinant. Nevertheless, we assume that the positions of centroids are not critical to successful classification. We replaced them by simple reference distribution approach. This is due to the classification refinement by the application of L{.} with $p_{L}$ choices, which represent the meta-optimization type settings.

#### 3.1.2. Classification which Converts the Original Time Series into Rényi Entropy Series

In analogy with the structure of the effective parameter $q_{\mathrm{TE}11}$ defined by Equation (8) we propose

(12) $$\alpha_{\mathrm{RE}}=\frac{\int_{\alpha_{m}}^{\alpha_{M}}d\alpha_{R}^{\prime }\;\alpha_{R}^{\prime }\;L\left\{S_{R}\right\}\left(p_{L},\alpha_{R}^{\prime }\right)}{\int_{\alpha_{m}}^{\alpha_{M}}d\alpha_{R}^{\prime \prime }\;L\left\{S_{R}\right\}\left(p_{L},\alpha_{R}^{\prime \prime }\right)}.$$

The scheme is built on Rényi entropy

(13) $$S_{R}\left(\alpha_{R}\right)=\frac{1}{1-\alpha_{R}}\log_{2}\left(\sum_{j=0}^{3}\pi_{j}^{\alpha_{R}}\right)$$

in which one parameter $\alpha_{R}>0$ is present. Similar to other applications we propose here, the values of $\alpha_{R}$ are delimited by the selection of the interval $[\alpha_{m},$ $\alpha_{M}]$. The averaging of the entropy series represented by $L\left\{S_{R}\right\}\left(p_{L},\alpha_{R}\right)$ is understood in the sense of Equation (5). Again, as in the case of $q_{\mathrm{TEyz}}$, two 1d integrations over $\alpha_{R}^{\prime }$ and $\alpha_{R}^{\prime \prime }$ are present in Equation (12). In agreement with previous minimalist implementation of integration rules we delimit ourselves to the ten function values that contribute to integration quadrature.

#### 3.1.3. Problem of Sarle’s b Revisited

In this subsection we revisit the problem of Sarle’s coefficient which standarly serves for diagnosing bimodality. In distinction to previous models we do not use probability distributions, but instead conditional local statistical averages which are constructed by

(14) $$\begin{array}{ccc} Skewness(T_{\mathrm{wdn}},T_{\mathrm{wup}}) & = & Arithm.Mean\left(\;z_{t}^{3}\;|\;t\in \left[T_{\mathrm{wdn}},T_{\mathrm{wup}}\right]\;\right)\;,\\ Kurtosis(T_{\mathrm{wdn}},T_{\mathrm{wup}}) & = & Arithm.Mean\left(\;z_{t}^{4}\;|t\in \left[T_{\mathrm{wdn}},T_{\mathrm{wup}}\right]\right) \end{array}$$

with auxiliary variable

(15) $$z_{t}=\frac{x_{t}-Aritm.Mean\left(\;x_{t}\;|\;t\in \left[T_{\mathrm{wdn}},T_{\mathrm{wup}}\right]\;\right)}{\sqrt{Var(\;x_{t}\;|\;t\in \left[T_{\mathrm{wdn}},T_{\mathrm{wup}}\right]\;)}}\;.$$

By combining Equation (14) terms, we get the interval (local) value

(16) $$b_{\mathrm{Sarle}}\left(T_{\mathrm{wdn}},T_{\mathrm{wup}}\right)=\frac{1+Skewness^{2}\;\left(T_{\mathrm{wdn}},T_{\mathrm{wup}}\right)}{Kurtosis(T_{\mathrm{wdn}},T_{\mathrm{wup}})}$$

applicable for ∀t∈ $[T_{\mathrm{wdn}},\;$ $T_{\mathrm{wup}}]$.

However, observations showed $b_{\mathrm{Sarle}}\left(.,.\right)$ sequence highly fluctuates in time within the samples. It implies that some generalized form of signal averaging is required to evaluate samples as a whole. Previous practice has indicated that we must be selective in dealing with fluctuations in the different signal parts. Thus, the Lehmer average $L\left(\left\{b_{\mathrm{Sarle}}\right\}\right)\left(p_{L}\right)$ over the $\{b_{\mathrm{Sarle}}(T_{\mathrm{wdn}},$ $T_{\mathrm{wup}})\}$ set of events is powerful option. With the selective averaging we obtained results depicted in Figure 5. They clearly explain why the original Sarle’s indicator (its value can be roughly associated with small $p_{L}$) is not sufficient for the classification and why the modification by means of the selective Lehmer weights play crucial role in the classification.

### 3.2. Integration over the $p_{L}$ Values-Option for t-Testing

We assume that it can be correctly expressed in the cumulative manner in which a particular number is assigned to each sample. To this end, for *j*-th sample we introduced the indicator

(17) $$I_{\mathrm{TEyz}}^{\left(j\right)}=\frac{1}{p_{M}-p_{m}}\;\int_{p_{m}}^{p_{M}}\;dp_{L}\;q_{\mathrm{TEyz}}^{\left(j\right)}\left(p_{L}\right)\;,\;j\in \mathrm{label}\left(A\right)\cup \mathrm{label}\left(B\right).$$

Here label(…) is the operator that assigns the respective label sets label(A), label(B) to the possible inputs A or B. The following comments on the above formula must be made: (**I**) No high precision integration over $p_{L}$ is required. The approximate tool for integral calculus we use is based on standard Riemann partitioning by means of 10 uniform rectangles per $\left[p_{m},p_{M}\right]$. It is important to note that it is not the precision of the integration itself, but the contribution to the level of deviations between the projections of A, B that matters most. (**II**) The integration boundaries $p_{m}$, $p_{M}$ should be properly chosen to include the negative relevant $p_{L}$. We used $p_{m}=-150$, $p_{M}=0$.

The selected statistical characteristics of $\left\{I_{\mathrm{TEyz}}^{\left(j\right)}\right\}$ for j∈label(A) and j∈label(B) are summarized in the Table 1. In all investigated cases of $T_{w}$, it was unexpectedly found that the average values of the numerical indicators $\left\{I_{\mathrm{TE}21}^{\left(j\right)}\right\}$, $\left\{I_{\mathrm{TE}22}^{\left(j\right)}\right\}$ showed higher relative medians (approximately 10 percent) when comparing A, B. This also indirectly points to the importance of introducing $w_{2}$ including the derivative of L{.} (see Equation (7)). However, the illustrative summary involved in Table 1 does not accurately represent the role of fluctuations.

In the Table 2, a statistically more accurate standard view is given. It presents statistical testing based on the two-sample *t*-values calculated using

(18) $$t_{\mathrm{AByz}}=\frac{Arithm.\;Mean\left(I_{\mathrm{ETyz}}^{\left(j\in \mathrm{label}(A)\right)}\right)-Arithm.\;Mean\left(I_{\mathrm{ETyz}}^{\left(j\in \mathrm{label}(B)\right)}\right)}{\sqrt{\frac{Var(I_{\mathrm{TEyz}}^{\left(j\in \mathrm{label}(A)\right)})}{\#A}+\frac{Var(I_{\mathrm{TEyz}}^{\left(j\in \mathrm{label}(B)\right)})}{\#B}}}\;,$$

where #A, #B are the respective cardinalities, while Var(…) stands for the unbiased variance. Therefore, by means of $I_{\mathrm{TEyz}}^{\left(j\right)}$ we use guidelines developed in the hypothesis testing. The degrees of freedom of *t*-distribution df are taken in the consistence with the standard Welch’s modified statistics [29,30]. Two samples, two sided *t*-tests for mean difference, the null hypotheses $t_{\mathrm{AByz}}=0$ are tested against $t_{\mathrm{AByz}}\neq 0$ alternatives. As a result, the significance of *p*-values supports the rejection of the null hypothesis tested in all four $I_{\mathrm{TEyz}}$ cases. Owing to the tendency to believe alternative hypotheses, the conclusions from the *t*-test are fully consistent with the classification proposed for A and B. The *t*-test is interestingly in some contrast with findings regarding the best practice for the choice of $I_{\mathrm{TE}y,z}^{\left(j\right)}$. The tests generally provide higher *t* for (y,z) ∈ {(1,1),(1,2)}. However, this result does not preclude the use of (y,z)∈ {(2,1),(2,2)} options, as the corresponding values of *t* remain very high in all situations.

## 4. Conclusions

While rich in information, single-molecule data are often heterogeneous and extensive. Additionally, the detection of rare and slow exchanging molecular states (category A) can be challenging due to interference with inactive, dormant states (category B). Here we have developed a specific supervised learning approach to address state classification problems in time-series data originating in a single molecule experiment. Our approach enables a clear identification of dormant molecular states and, hence, it makes statistical evaluations possible. Once statistical evaluation is performed, the analysis can proceed further to evaluate and characterize rare molecular states. While our particular method, where entropy is an important component of the evaluation, has shown progress, it can be further developed in a variety of directions.

For example, the additional goal and the next step might be to optimize the efficiency of categorization. Thanks to the outcomes of the statistical tests, *t*-values can be used as an optimization criterion. In this respect, there may be different choices for $w_{1}$, $w_{2}$, which may cause variations in the efficiency of the separation of A from B time series classes. Thus, the next goal also be to concentrate more systematically on the function spaces generated by $w_{1}$, $w_{2}$ arguments.

The comparison of several methodological variants shows that Lehmer averaging has a much deeper impact on results than we originally expected. The optimality of the classification may come from different sources and effects, which is also confirmed by the fact that it manifests itself in different areas of the control parameter $p_{L}$.

Using the transition probabilities for a sequence of stable molecular states, one can systematically explore the potential of the entropy-based approach. For example, a transition study will certainly offer a new perspective on updating the classification. Furthermore, adaptive conditional averages used herein can improve the manner of discriminating the state of space. These inputs can be implemented by HMM Viterbi’s method, which is considered standard in today’s analysis. Hence, our new conceptual framework can further enhance an in-depth understanding of the dynamics of individual molecules.

## Figures and Tables

**Table 1:** Summary of the descriptive characteristics of the system of samples. The evaluation performed by means of R generic function summary(.) [29]. The respective averages calculated $\left\{I_{\mathrm{TEyz}}^{\left(j\right)}\right\}$, j∈{A,B} with $I_{\mathrm{TEyz}}^{\left(j\right)}$, defined by Equation (17). In line with the previous considerations we deal with the three selected values of $T_{w}$. The differences in the corresponding values of A and B in the respective columns Min, 1st Qu, …, Max. (Note that 1st Qu means first quartile, while 3rd Qu is the label of the third quartile of observations.) All A items are larger than B, indicating observable separability at different time window sizes. For example, greater inter-group changes might indicate a better contrast in distinguishing between classes A and B. For clarity, the items where the relative median changes exceed 10 percent are marked with an asterisk ${★}_{>10\%}$. In such cases the corresponding rather strongly varying indicators $I_{\mathrm{TE}21}$, $I_{\mathrm{TE}22}$ are marked in blue. More passive tendency regarding changes is labeled by the circles $\circ_{<2\%}$.

Window	Category	Indicator	Relative:	Min	1st Qu	Median	Mean	3rd Qu	Max
$T_{w}$	Samples		Median for A to Median for B	$\left\{I_{\mathrm{TEyz}}^{\left(j\right)}\right\}$	$\left\{I_{\mathrm{TEyz}}^{\left(j\right)}\right\}$	$\left\{I_{\mathrm{TEyz}}^{\left(j\right)}\right\}$	$\left\{I_{\mathrm{TEyz}}^{\left(j\right)}\right\}$	$\left\{I_{\mathrm{TEyz}}^{\left(j\right)}\right\}$	$\left\{I_{\mathrm{TEyz}}^{\left(j\right)}\right\}$
500	A	$I_{\mathrm{TE}11}$	$\circ_{<2\%}$	2.577	2.586	2.590	2.592	2.599	2.627
500	B	$I_{\mathrm{TE}11}$	$\circ_{<2\%}$	2.558	2.559	2.559	2.560	2.560	2.565
500	A	$I_{\mathrm{TE}12}$	$\circ_{<2\%}$	2.971	2.980	2.984	2.986	2.994	3.021
500	B	$I_{\mathrm{TE}12}$	$\circ_{<2\%}$	2.953	2.954	2.954	2.954	2.954	2.959
500	A	$I_{\mathrm{TE}21}$	${★}_{>10\%}$	1.607	1.718	1.801	1.828	1.894	2.386
500	B	$I_{\mathrm{TE}21}$	${★}_{>10\%}$	1.427	1.511	1.522	1.520	1.541	1.629
500	A	$I_{\mathrm{TE}22}$	${★}_{>10\%}$	1.714	1.835	1.928	1.947	2.013	2.466
500	B	$I_{\mathrm{TE}22}$	${★}_{>10\%}$	1.524	1.623	1.636	1.633	1.658	1.749
1000	A	$I_{\mathrm{TE}11}$	$\circ_{<2\%}$	2.583	2.589	2.595	2.597	2.601	2.628
1000	B	$I_{\mathrm{TE}11}$	$\circ_{<2\%}$	2.554	2.555	2.556	2.556	2.556	2.559
1000	A	$I_{\mathrm{TE}12}$	$\circ_{<2\%}$	2.978	2.984	2.991	2.991	2.996	3.023
1000	B	$I_{\mathrm{TE}12}$	$\circ_{<2\%}$	2.949	2.950	2.950	2.950	2.951	2.954
1000	A	$I_{\mathrm{TE}21}$	${★}_{>10\%}$	1.735	1.818	1.918	1.940	2.020	2.473
1000	B	$I_{\mathrm{TE}21}$	${★}_{>10\%}$	1.476	1.542	1.567	1.558	1.578	1.589
1000	A	$I_{\mathrm{TE}22}$	${★}_{>10\%}$	1.867	1.944	2.050	2.069	2.135	2.576
1000	B	$I_{\mathrm{TE}22}$	${★}_{>10\%}$	1.584	1.660	1.689	1.679	1.704	1.716
2000	A	$I_{\mathrm{TE}11}$	$\circ_{<2\%}$	2.579	2.588	2.591	2.592	2.595	2.608
2000	B	$I_{\mathrm{TE}11}$	$\circ_{<2\%}$	2.553	2.554	2.554	2.554	2.555	2.556
2000	A	$I_{\mathrm{TE}12}$	$\circ_{<2\%}$	2.973	2.982	2.986	2.986	2.991	3.002
2000	B	$I_{\mathrm{TE}12}$	$\circ_{<2\%}$	2.948	2.949	2.949	2.949	2.949	2.951
2000	A	$I_{\mathrm{TE}21}$	${★}_{>10\%}$	1.711	1.787	1.834	1.867	1.933	2.220
2000	B	$I_{\mathrm{TE}21}$	${★}_{>10\%}$	1.564	1.577	1.583	1.584	1.590	1.601
2000	A	$I_{\mathrm{TE}22}$	${★}_{>10\%}$	1.816	1.929	1.985	2.012	2.090	2.328
2000	B	$I_{\mathrm{TE}22}$	${★}_{>10\%}$	1.686	1.703	1.710	1.711	1.719	1.733

**Table 2:** Comparison of A, B projections of the type $I_{\ldots }^{\left(j\right)}$ quantified in terms of *t*-statistics. Calculated for four types of $I_{\mathrm{TEyz}}^{\left(j\right)}$ with the variants (y,z)∈{(1,1); (1,2); (2,1); (2,2)}. The effective number of degrees of freedom df is calculated, which represents the input of Student’s t distribution function. Accordingly, these sufficiently small *p*-values imply the rejecting of H0: $t_{\mathrm{AByz}}=0$.

$T_{w}$	(y,z)	$t_{\mathrm{AByz}}$	df	*p*-Value	95% Confidence Interval
500	(1,1)	17.583	33.058	2.409 × $10^{-18}$	[2.559,2.591]
500	(1,2)	17.101	32.944	5.987 × $10^{-18}$	[2.954,2.986]
500	(2,1)	10.260	34.032	5.945 × $10^{-12}$	[1.519,1.828]
500	(2,2)	10.520	34.998	2.213 × $10^{-12}$	[1.633,1.946]
1000	(1,1)	25.059	31.799	$1.652\times 10^{-22}$	[2.555,2.596]
1000	(1,2)	24.708	31.770	$2.609\times 10^{-22}$	[2.950,2.991]
1000	(2,1)	13.513	32.666	$6.301\times 10^{-15}$	[1.557,1.939]
1000	(2,2)	14.297	33.564	7.781 × $10^{-16}$	[1.679,2.069]
2000	(1,1)	32.646	31.643	6.133 × $10^{-26}$	[2.555,2.591]
2000	(1,2)	31.538	31.636	1.786 × $10^{-25}$	[2.948,2.986]
2000	(2,1)	13.536	31.419	$1.178\times 10^{-14}$	[1.583,1.867]
2000	(2,2)	14.365	31.654	2.041 × $10^{-15}$	[1.711,2.012]
